# Carbon Dioxide Inhalation—Risks for Health or Opportunity for Physical Fitness Development?

**DOI:** 10.3390/jcm15010364

**Published:** 2026-01-03

**Authors:** Natalia Danek

**Affiliations:** Department of Physiology and Biomechanics, Faculty of Physical Education and Sport, Wroclaw University of Health and Sport Sciences, 51-612 Wroclaw, Poland; natalia.danek@awf.wroc.pl; Tel.: +48-713473358

**Keywords:** hypercapnia, CO_2_ inhalation, respiratory dead space, voluntary hypoventilation, exercise performance, buffering capacity, sports physiology

## Abstract

**Background**: Carbon dioxide (CO_2_) is traditionally regarded as a metabolic by-product; however, growing evidence indicates that it plays an active regulatory role across multiple physiological systems. Acute hypercapnia elicits respiratory, cardiovascular, metabolic, immune, and neurocognitive responses, some of which may transiently influence exercise performance. This narrative review summarizes current evidence on CO_2_ inhalation in healthy individuals and critically evaluates whether controlled hypercapnia may serve as a targeted stimulus in sport and exercise contexts. **Methods**: A narrative review of peer-reviewed English-language articles indexed in PubMed and Web of Science was conducted. A narrative approach was chosen due to the marked heterogeneity of study designs, hypercapnia-induction methods (e.g., CO_2_ inhalation, voluntary hypoventilation, increased respiratory dead space), participant characteristics, and outcome measures, which precluded systematic synthesis. The review focused on studies involving healthy or physically active individuals and examined acute or short-term hypercapnic exposure. No strict publication date limits were applied. Studies conducted exclusively in clinical populations were excluded. **Results**: Short-term, controlled hypercapnia reliably increases ventilation, sympathetic activation, cerebral and muscular blood flow, and metabolic stress. Certain hypercapnia-based interventions—such as voluntary hypoventilation or added respiratory dead space—may enhance buffering capacity, reduce lactate accumulation and improve maximal oxygen uptake (VO_2_max) during submaximal efforts and repeated-sprint performance during high-intensity, short-duration exercise. However, CO_2_ inhalation frequently induces dyspnea, anxiety, and cognitive disruption, and higher concentrations pose clear safety risks. Current evidence does not support long-term improvements in VO_2_max or long-duration endurance performance following hypercapnia-based interventions. **Conclusions**: Controlled, intermittent hypercapnia may provide a targeted metabolic and ventilatory stimulus that enhances tolerance to high-intensity exercise, yet its application remains experimental and context-dependent. The risks associated with CO_2_ inhalation in healthy individuals currently outweigh its potential benefits, and safe, effective training protocols have not been fully established. Further research is needed to clarify the mechanisms, long-term adaptations, and practical utility of hypercapnia-based training strategies.

## 1. Introduction

Inhalation of carbon dioxide (CO_2_)-enriched air increases its partial pressure in arterial blood above 45 mmHg, leading to hypercapnia. Hypercapnia is defined as a condition in which the arterialized blood partial pressure of carbon dioxide (pCO_2_) exceeds 45 mmHg [1]. When the elevation in CO_2_ partial pressure is accompanied by increased carbonic acid (H_2_CO_3_^−^) concentration and a reduction in pH (reflecting elevated hydrogen ion concentration) [2], a state of respiratory (hypercapnic) acidosis develops. Carbonic acid forms as a result of the reaction between CO_2_ and water (H_2_O) and, under the catalytic action of carbonic anhydrase (CA), dissociates reversibly into H^+^ and HCO_3_^−^.

Hypercapnia is a potent stimulus for the respiratory center located in the brainstem. Even a small rise in CO_2_ above atmospheric levels (~0.04%) triggers reflex hyperventilation—an increase in breathing rate and depth—as a compensatory mechanism to eliminate excess CO_2_ from the body [3]. This response is mediated by respiratory chemoreceptors: central chemoreceptors in the medulla, which detect decreases in cerebrospinal fluid pH, and peripheral chemoreceptors in the carotid and aortic bodies, which respond to reductions in arterial pH associated with elevated pCO_2_ [3]. Consequently, even moderate elevations in inspired CO_2_ produce a measurable increase in minute ventilation. Moreover, hypercapnia shifts the acid–base balance toward respiratory acidosis, with rising hydrogen ion concentration further stimulating the respiratory drive.

Carbon dioxide is continuously produced in the tricarboxylic acid cycle as the principal by-product of aerobic metabolism. In the bloodstream, it is transported in three major forms: approximately 80% as bicarbonate, 5–10% physically dissolved in plasma, and 10–15% bound as carbaminohemoglobin [4]. The CO_2_ content of expired air is about 5%, several hundred times higher than in inspired ambient air (~0.03%) [5]. Inhalation of CO_2_-enriched air therefore induces almost immediate alterations in the respiratory, cardiovascular, and nervous systems. CO_2_ is a crucial metabolic gas that plays a key role in numerous physiological processes, including cellular respiration, modulation of hemoglobin–oxygen affinity, and the maintenance of acid–base homeostasis and blood pH regulation [1]. It also exhibits notable vasodilatory and immunomodulatory properties [6]. Due to its small molecular size, CO_2_ readily diffuses across cell membranes down its transmembrane concentration gradient, influenced by the lipid–water partition coefficient. Once inside the cell, it undergoes hydration to form carbonic acid (H_2_CO_3_), which subsequently dissociates into hydrogen (H^+^) and bicarbonate (HCO_3_^−^) ions. This process is highly accelerated by carbonic anhydrase [5]. In healthy individuals, CO_2_ concentrations are maintained within a narrow range by feedback mechanisms involving both central and peripheral chemoreceptors [7,8]. The mechanical compliance of the thoracic cage determines pulmonary minute ventilation; thus, even with normal respiratory center activity and intact respiratory muscle function, mechanical limitations of the lungs may occur [9]. As a result, compensatory increases in respiratory rate (RR) may develop, contributing to fatigue while failing to sufficiently enhance ventilation to eliminate excess CO_2_, ultimately leading to hypercapnia [10]. These relationships underscore that respiratory rate (RR) and tidal volume (VT) are the primary determinants of ventilation, regulated by central and peripheral chemoreceptors. They can be modulated physiologically or adjusted artificially to maintain optimal CO_2_ elimination under both physiological and therapeutic conditions [11].

This narrative review synthesizes peer-reviewed English-language publications, including original research articles and review papers indexed in databases such as PubMed and Web of Science. The literature was selected based on relevance to carbon dioxide (CO_2_) inhalation and its physiological and performance-related effects in healthy individuals. Particular emphasis was placed on studies examining acute CO_2_ exposure and interventions that induce hypercapnia—such as voluntary hypoventilation and respiratory dead space loading—with a focus on physiological responses, exercise performance outcomes, and potential training adaptations in physically active individuals and athletes. This review therefore aims to inform practitioners, coaches, athletes, and researchers about the possible effects, challenges, and potential applications of CO_2_ inhalation methods, as well as the necessary precautions required when implementing them.

To ensure conceptual clarity, it is important to distinguish between the different approaches through which hypercapnia may be induced in exercise and training contexts. In this review, CO_2_ inhalation refers specifically to the breathing of gas mixtures enriched with carbon dioxide (e.g., carbogen), in which hypercapnia results directly from an increased inspired CO_2_ fraction. In contrast, hypercapnia-inducing methods represent a broader category that also includes techniques not involving external CO_2_ enrichment, such as CO_2_ rebreathing via increased respiratory dead space (e.g., masks or tubing systems that promote partial re-inhalation of expired air) and voluntary hypoventilation or breath-hold-based methods, in which hypercapnia arises secondary to reduced alveolar ventilation. Importantly, these approaches differ substantially in their physiological mechanisms and safety profiles; for example, voluntary hypoventilation is frequently accompanied by concomitant hypoxemia, whereas CO_2_ inhalation primarily alters arterial pCO_2_ without necessarily reducing oxygen availability. Throughout this manuscript, the term hypercapnia-inducing methods is therefore used as an umbrella concept, while more specific terminology is applied when referring to individual mechanisms, in order to accurately reflect their distinct physiological and practical implications.

## 2. Materials and Methods

Narrative reviews play an important role in advancing knowledge not only by summarizing existing evidence, but also by contextualizing how and why specific phenomena have been studied, how key concepts have been defined and interpreted over time, and how different lines of evidence inform current research and applied practice. In the present study, we conducted a narrative review to synthesize and critically interpret the literature on hypercapnia and CO_2_-based interventions in the context of exercise physiology and performance. The literature search was performed using PubMed and Web of Science, employing a keyword-based strategy. Articles were identified when their titles or abstracts contained one or more of the following terms or combinations thereof: hypercapnia, hypercapnic training, carbogen, CO_2_ inhalation, dead space, rebreathing, voluntary hypoventilation, breath-hold training, and hypercapnic warm-up. Reference lists of relevant articles were also screened to identify additional studies using a snowballing approach. International, peer-reviewed articles published in English were considered for inclusion. The review focused on studies involving healthy individuals, physically active participants, or athletes, examining acute or short-term hypercapnic exposure at rest or during exercise. Studies conducted exclusively in clinical populations were excluded. No strict temporal limits were applied, as several foundational experimental and physiological studies published in the 1970s and 1980s remain central to the mechanistic understanding of hypercapnia-related ventilatory, metabolic, and vascular responses. Older studies were included only when their methodology and conclusions were consistent with and supported by more recent evidence. Each identified record was assessed for relevance by the authors, and studies were selected based on their contribution to understanding the physiological and performance-related effects of hypercapnia. This review is not intended to be exhaustive, but rather to provide an integrative synthesis of key concepts, mechanisms, and experimental findings that underpin current research and potential applications of hypercapnia-based methods in exercise and sport.

## 3. Results

### 3.1. Neuroendocrine Responses to Acute and Chronic Hypercapnia

An increase in the concentration of CO_2_ in inspired air exerts a strong effect on the nervous system. Short-term inhalation of high CO_2_ levels (above approximately 5–7%) induces symptoms such as dizziness, disorientation, visual disturbances, and a sense of mental clouding. These effects result from the impact of CO_2_ on the central nervous system—elevated CO_2_ levels (hypercapnia) cause cerebral vasodilation and an increase in intracranial pressure, which manifest clinically as neurological symptoms, including headache and confusion. At the same time, CO_2_ powerfully stimulates the respiratory center and the sympathetic nervous system, triggering so-called “respiratory stress.” In healthy individuals, even a slight rise in arterial pCO_2_ rapidly activates a classical “fight-or-flight” response, leading to the release of adrenaline and noradrenaline, increased heart rate and respiratory rate, and elevated blood pressure. Consequently, short-term CO_2_ inhalation produces a subjective feeling of intense arousal, anxiety, and muscular tension, often accompanied by somatic manifestations such as trembling, sweating, and tachycardia. From an endocrine standpoint, CO_2_ acts as a stressor that activates the hypothalamic–pituitary–adrenal (HPA) axis. For example, some studies have reported increased cortisol concentrations following a 35% CO_2_ challenge, although findings have not always been consistent [12].

During chronic hypercapnia, the body initiates compensatory mechanisms. The kidneys increase bicarbonate (HCO_3_^−^) reabsorption and hydrogen ion excretion to buffer respiratory acidosis. As a result, circulating bicarbonate levels rise and acid–base balance is gradually restored, albeit at the expense of metabolic alterations. This adaptation allows survival under persistently elevated pCO_2_ but may carry physiological costs. Prolonged acidosis may, for instance, promote bone mineral loss, as calcium and phosphate ions are mobilized to buffer excess hydrogen ions. The literature indicates that CO_2_ concentrations exceeding 10,000 ppm (1%) can lead to measurable metabolic disturbances, including reductions in ionized calcium levels and increased urinary phosphate excretion [12,13,14,15,16,17,18]. Furthermore, sustained hypercapnia-driven activation of the HPA axis may dysregulate endocrine function, with chronically elevated cortisol and catecholamines contributing to disrupted sleep and impaired glucose homeostasis [15]. Importantly, no direct long-term benefits of CO_2_ inhalation for neuromuscular or endocrine function in healthy individuals have been demonstrated; chronic hypercapnia is generally regarded as a detrimental physiological state.

### 3.2. Metabolic and Endocrine Adaptations to Hypercapnia

Carbon dioxide is closely linked to metabolic processes, as it represents the final product of carbohydrate and fat oxidation, and its concentration in the blood (pCO_2_) directly influences the acid–base balance of the body. Short-term inhalation of CO_2_-containing gas mixtures leads to a rapid increase in arterial pCO_2_, resulting in acute respiratory acidosis, which is characterized by an immediate decrease in blood pH [18].

Experimental studies have shown that exposure to approximately 1% CO_2_ (≈10,000 ppm) can cause metabolic disturbances associated with compensation for acidosis. The review by Azuma et al. [10] indicates that CO_2_ concentrations above 10,000 ppm in indoor environments are linked to markers of metabolic stress, such as reductions in ionized calcium levels and increased urinary phosphate excretion (Table 1). Similar biochemical alterations have been reported in reviews examining the effects of acidosis on calcium–phosphate homeostasis, in which a decrease in pH promotes the mobilization of bone and renal buffers [16]. An acute rise in CO_2_ also acts as a physiological stressor. Human experimental studies have demonstrated that hypercapnia activates the hypothalamic–pituitary–adrenal (HPA) axis and the sympathetic nervous system, leading to increased circulating cortisol, adrenaline, and noradrenaline levels [11,12,16,17]. From a metabolic perspective, this response contributes to elevated blood glucose, enhanced lipolysis, and a general mobilization of energy substrates. CO_2_ inhalation can therefore be considered a metabolic stress stimulus.

At the same time, moderate increases in pCO_2_ induce predictable physiological effects associated with the Bohr effect: elevated pCO_2_ and reduced pH facilitate the release of oxygen from hemoglobin to tissues [15]. This mechanism is utilized in gas mixtures containing 5% CO_2_, widely known as carbogen (5% CO_2_ + 95% O_2_). Research has shown that short-term exposure to 5% CO_2_ is well tolerated and does not cause systemic adverse effects in healthy individuals [18]. Carbogen is commonly employed in diagnostic procedures, such as assessments of cerebrovascular reactivity, in which elevated pCO_2_ induces robust cerebral vasodilation and increases cerebral blood flow, enabling evaluation of vascular responsiveness using MRI or fMRI techniques [19]. From a metabolic standpoint, short-term, moderate hypercapnia may transiently enhance oxygen and substrate delivery to tissues due to both the Bohr effect and increased blood perfusion.

### 3.3. Cognitive and Psychological Responses to Acute and Chronic Hypercapnia

Carbon dioxide has a unique ability to evoke strong anxiety and panic responses in humans. This effect is so pronounced that CO_2_ inhalation has become an established experimental model in psychiatry, widely known as the CO_2_ challenge test, used to induce panic-like symptoms. Two main protocols are commonly employed: a single deep inhalation of a gas mixture containing 35% CO_2_, or several minutes of inhalation of 7–7.5% CO_2_ in ambient air. In healthy volunteers, such exposures reliably induce intense anxiety, a sensation of “air hunger,” and somatic symptoms including tachycardia, chest discomfort, and dizziness. Numerous studies consistently demonstrate that CO_2_ inhalation elicits an anxiety response closely resembling a panic attack—subjectively similar to spontaneous panic episodes described by patients. Individuals with panic disorder or post-traumatic stress disorder (PTSD) typically exhibit even stronger responses to CO_2_. For example, studies show that inhalation of 35% CO_2_ in patients with PTSD, compared with placebo gas, triggers significantly higher levels of panic, anxiety, and dissociative symptoms, as well as trauma-related intrusive memories [20,21]. Importantly, healthy individuals rarely experience a full panic attack during such tests, whereas most patients with panic disorder do, making CO_2_ reactivity a diagnostic marker [22]. Research also indicates that first-degree relatives of individuals with panic disorder are more likely to panic during CO_2_ exposure, with a three-fold higher relative risk compared with the general population [21]. This suggests that CO_2_ sensitivity may represent a heritable vulnerability factor for anxiety disorders. The underlying mechanisms are not fully understood, but it is commonly hypothesized that specialized chemosensory neurons in the brain detect increases in CO_2_ or decreases in pH and trigger a primordial fear response—the so-called “suffocation alarm.” Supporting this hypothesis, Griez et al. [21] showed that inhalation of 7.5% CO_2_ in healthy individuals heightened hypervigilance, increasing attentional bias toward threat-related stimuli and overall alertness and orienting responses. In other words, CO_2_ induces a state of heightened readiness and anxiety-driven vigilance, which is evolutionarily consistent with detecting suffocation threats. However, heightened anxiety may impair higher-order cognitive functions. Diaper et al. [23] demonstrated that while simple manual tasks were performed slightly better under 7.5% CO_2_ (likely due to narrowed attentional focus), performance on more complex cognitive tasks deteriorated significantly, with increased subjective workload and reduced efficiency. Thus, mild anxiety may sometimes improve focus, but high levels of CO_2_-induced anxiety impair cognitive performance.

### 3.4. Immune System Responses to Acute and Chronic Hypercapnia

The effects of CO_2_ on the immune system are complex and remain an active area of investigation. On the one hand, hypercapnia may initiate inflammatory processes; on the other, it can exert immunosuppressive or anti-inflammatory effects depending on the context. Short-term inhalation of pure CO_2_ (up to approximately 5–7%) in healthy individuals does not induce a systemic inflammatory response—no fever or significant changes in blood morphology have been observed following a few minutes of exposure, as confirmed by volunteer studies [24]. However, localized alterations within tissues may occur. Conversely, numerous studies point to an immunosuppressive action of CO_2_. Elevated pCO_2_ has been shown to suppress various immune functions. At the molecular level, hypercapnia (20% CO_2_ for 24 h) alters the expression of hundreds of genes in airway epithelial cells, with many immune-related genes significantly downregulated [25]. Infection models also show that high CO_2_ can impair host defense: it reduces the activity of neutrophils and pulmonary macrophages, diminishing their capacity for phagocytosis and bacterial killing [6,26,27]. Consequently, animals kept in a CO_2_-enriched atmosphere exhibit poorer bacterial clearance from the lungs. In studies of pneumonia, mice exposed to 10% CO_2_ had worse outcomes than those breathing normal air, with higher mortality due to weakened innate immune responses. In summary, current evidence indicates that short-term, moderate hypercapnia may exert anti-inflammatory effects by attenuating excessive inflammatory responses; however, prolonged exposure to elevated CO_2_ appears to be predominantly immunosuppressive, compromising innate immunity.

### 3.5. Cardiorespiratory Responses to Acute and Chronic Hypercapnia

In healthy individuals, small increases in ambient CO_2_ concentration (0.05–0.5%, i.e., 500–5000 ppm) induce linear and gradual physiological changes in the cardiovascular and autonomic systems—such as increased respiratory rate and heart rate—although they typically do not cause pronounced subjective symptoms [28,29]. Carbon dioxide exerts a vasodilatory effect, particularly within the cerebral circulation, resulting in increased cerebral blood flow even during mild hypercapnia in healthy volunteers [30]. Conversely, more substantial hypercapnia stimulates the sympathetic nervous system. During inhalation of 7.5% CO_2_, clear increases in heart rate and arterial blood pressure are observed, indicating generalized adrenergic activation [31]. Healthy individuals usually tolerate brief exposures to CO_2_ concentrations up to approximately 5% without major physiological complications [28]. However, higher concentrations (>7–10%) can induce dangerous symptoms, including cardiac arrhythmias and significant fluctuations in blood pressure, especially during prolonged exposure. Early symptoms of hypercapnia include dyspnea, tachypnea (hyperventilation), peripheral vasodilation (causing warm, flushed skin), sweating, and a fuller pulse. As arterial pCO_2_ rises above normal levels, headaches and dizziness may appear, partly due to increased intracranial pressure resulting from cerebral vasodilation [29,30]. With respect to the respiratory system, hypercapnia directly stimulates ventilation. During inhalation of gas mixtures enriched with CO_2_, healthy individuals show an immediate increase in both respiratory rate and tidal volume. This effect is mediated by chemoreceptors—an increase in CO_2_ partial pressure of only a few mmHg above normal markedly elevates minute ventilation. Studies have shown that adding 2–4% CO_2_ to inspired air produces a gradual, proportional increase in pulmonary ventilation [28]. This hyperventilatory response constitutes a protective mechanism against progressive respiratory acidosis: by expelling excess CO_2_ through intensified ventilation, the body attempts to restore normal blood pH. CO_2_ is the primary driver of ventilation, as respiratory control is governed mainly by CO_2_ levels. Therefore, CO_2_ inhalation exerts immediate effects on respiratory function. Short-term inhalation of a CO_2_-rich mixture evokes rapid, deep breathing (hyperventilation). For example, inhalation of 7% CO_2_ induces a pronounced sensation of air hunger within seconds, because brainstem chemoreceptors detect elevated CO_2_ and initiate maximal ventilatory drive. Subjectively, this manifests as intense breathlessness. Breathing becomes fast and deep, which may paradoxically reduce CO_2_ levels through hyperventilation. This process is accompanied by tachycardia and often increased arterial blood pressure.

The cardiovascular system responds to CO_2_ is bidirectional and context dependent. Sympathetic activation mediated by the vasomotor center causes vasoconstriction of many peripheral vessels and increases blood pressure, while on the other hand, CO_2_ exerts a direct vasodilatory effect—most prominently in the cerebral, coronary, and skeletal muscle circulations—leading to increased regional perfusion. Under conditions of isolated, moderate hypercapnia (typically <8% CO_2_, with preserved oxygen availability), the net effect is usually a modest rise in systolic and diastolic blood pressure by several mmHg, without immediate life-threatening consequences. At higher concentrations (>10% CO_2_), however, the risk profile changes substantially, particularly when hypercapnia is accompanied by a reduction in inspired oxygen fraction. In such mixed scenarios (hypercapnia plus hypoxia), acidosis and tissue hypoxia jointly impair myocardial electrical conduction, increasing susceptibility to cardiac arrhythmias. Clinical manifestations may include chest pain, palpitations, and—under extreme conditions—life-threatening rhythm disturbances such as ventricular fibrillation. Experimental studies show that ~5% CO_2_ causes dizziness, fatigue, dyspnea, and mild headache in healthy individuals [28]. Concentrations of 8–10% induce pronounced neurological symptoms, including severe headache, confusion, sweating, visual disturbances, and extreme ventilatory distress. Inhalation of such concentrations for several minutes approaches human tolerance limits, often producing gagging reflexes, panic, and potentially loss of consciousness due to hypercapnic narcosis. At this concentration, CO_2_ poses a severe risk, because loss of consciousness can compromise effective ventilation and precipitate secondary hypoxia [32].Despite these alarming effects, moderate hypercapnia under controlled, oxygen-replete conditions can be used safely under controlled conditions for specific physiological benefits. A well-established example is the use of 5% CO_2_ (with supplemental oxygen) in medicine. As noted earlier, this mixture—carbogen—is considered safe during short exposures, with studies involving hundreds of participants showing no harmful consequences of breathing 5% CO_2_ for several minutes [28,30]. On the contrary, it elicits controlled effects useful for diagnostic purposes by inducing vasodilation in cerebral and coronary vessels, enabling assessment of vascular reserve in the brain or heart. Due to its vasodilatory properties, CO_2_ improves tissue perfusion and oxygenation; for instance, healthy individuals breathing 5% CO_2_ exhibit an approximately 20–30% increase in mean cerebral blood flow [29]. Increased peripheral flow has also been observed, as CO_2_ dilates cutaneous and muscular vessels. This mechanism is applied in balneotherapy: immersion in CO_2_-rich water improves blood flow to the extremities and supports treatment of peripheral vascular disease [32]. In addition, CO_2_ acts as a potent respiratory stimulant. Historically, 5% CO_2_ was used to activate respiration in neonates and patients with sleep apnea, and controlled elevations of inspired CO_2_ are still employed during emergence from anesthesia to facilitate the return of spontaneous breathing [32]. Mild hypercapnia may further improve oxygenation by dilating small bronchioles and pulmonary vessels, thereby enhancing ventilation–perfusion matching and promoting alveolar fluid clearance in conditions such as pulmonary edema [32].

**Figure 1:**
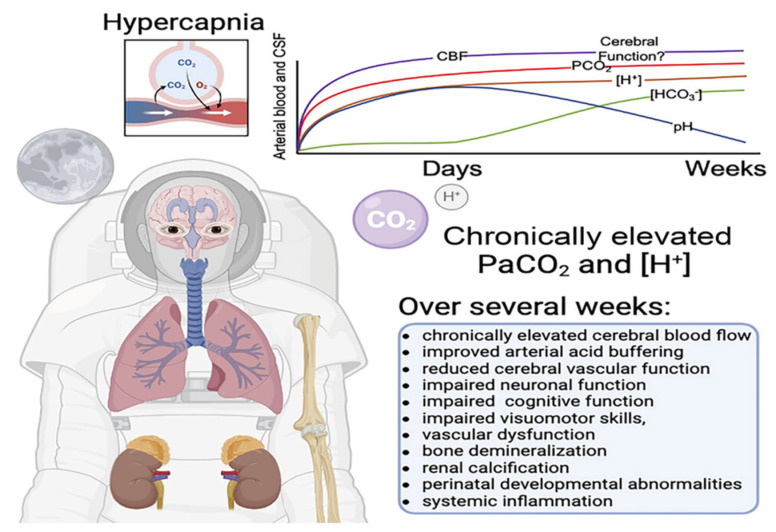
Cerebral implications of chronic elevations of inspired carbon dioxide—source from Carr et al. [14].

The long-term effects of cardiorespiratory exposure to CO_2_ are relevant in environmental and occupational settings. Workers in enclosed environments (submarines, space stations, mines) may be chronically exposed to CO_2_ levels around 0.5–1% (Figure 1). Studies indicate that prolonged inhalation of ~0.5% CO_2_ results in physiological adaptation but also symptoms such as persistent headache, fatigue, impaired concentration, and elevated blood pressure [33]. Epidemiological data show that CO_2_ concentrations above 1000 ppm in indoor environments correlate with increased respiratory irritation and symptoms such as coughing [34]. In individuals with pre-existing pulmonary disease, chronic hypercapnia can induce structural changes in the pulmonary circulation: elevated pCO_2_ (often coexisting with hypoxia) causes pulmonary vasoconstriction and, over time, pulmonary hypertension. This increases cardiac workload and contributes to right ventricular hypertrophy [35]. Despite these concerns, controlled hypercapnic acidosis offers protective effects, including reduced lung injury [36].

## 4. Applications of CO_2_ Inhalation in Sports Training

The induction of hypercapnia elicits a sympathetic response that increases pulmonary minute ventilation (VE) through coordinated modulation of tidal volume (VT) and respiratory frequency (fR). The ventilatory response is determined by central and peripheral chemoreceptors that are sensitive to changes in arterial pO_2_, pCO_2_ and hydrogen ion concentration (H^+^). Central chemoreceptors, located within chemosensitive regions of the brainstem, respond predominantly to changes in cerebrospinal fluid pH resulting from elevations in arterial pCO_2_. Their activation stimulates the respiratory center, producing both deeper and faster breathing. Peripheral chemoreceptors—primarily situated in the carotid bodies and, to a lesser extent, in the aortic bodies—are uniquely sensitive to arterial hypoxemia but also respond dynamically to changes in pCO_2_ and hydrogen ion concentration. Importantly, peripheral chemoreceptors exhibit rapid responsiveness to the rate of change in the chemical stimulus rather than its absolute magnitude, which makes them particularly relevant during exercise and other non-steady-state conditions [37,38]. Chemosensitivity, defined as the ventilatory response to hypoxia or hypercapnia, is modulated by several factors, including body temperature [39], hormonal status, and genetic predisposition, contributing to substantial interindividual variability in ventilatory responses to elevated CO_2_. Under resting physiological conditions, central chemoreceptors exert a dominant influence on ventilation; however, during exercise or hypoxic exposure, the relative contribution of peripheral chemoreceptors increases [40,41]. This shift is especially relevant in hypercapnic or hypoxic exercise scenarios, where rapid ventilatory adjustments are required to stabilize acid–base balance. Ohkuwa et al. [42] observed greater ventilatory responses to changes in CO_2_ in sprinters compared with endurance swimmers. With training, chemoreflex sensitivity to changes in pCO_2_ decreases, indicating adaptive processes within the respiratory control system [43]. Carbon dioxide, as the main factor modulating chemosensitivity, also influences vascular interactions in the body, including cardiac mechanical function. In the study by Kato et al. [44], breathing a gas mixture containing 6% CO_2_ during a progressive exercise test resulted in significantly higher heart rate values during recovery compared with standard breathing conditions. Similarly, in an experiment in which participants breathed air with 5% CO_2_ during continuous exercise at 30% and 60% VO_2_max, higher heart rate values were observed [45]. In contrast, Jones et al. [46] did not report differences in heart rate during continuous exercise at 33%, 66%, and 95% VO_2_max under conditions of varying inspired CO_2_ concentration. Numerous studies indicate that increased CO_2_ levels lead to vasodilation and an increase in blood flow velocity in the middle cerebral artery, thereby enhancing cerebral perfusion [39,47,48]. Regulation of the partial pressures of oxygen and carbon dioxide requires ventilatory activity adjusted to exercise intensity so as to maintain pCO_2_ within a tolerable physiological range [49]. Furthermore, ventilation must be regulated to prevent the development of either respiratory acidosis or alkalosis [49,50,51]. An increase in VE is proportional to the additional dead space volume [49,52,53]. The rise in VE induced by devices that increase dead space may depend on both an increase in VT and changes in fR. In the study by McParland et al. [54], the application of 940 mL of additional dead space during a progressive test altered the composition of VE at 120 L·min^−1^, increasing VT from 2.9 to 3.3 L and decreasing fR from 41 to 36 breaths per minute, compared with conditions without added respiratory load. In contrast, in our own research [55], in which an ARDSv device with a volume of 1200 mL was used during interval exercise, we observed increases in both VT and fR. These differences may result from variations in exercise characteristics as well as the magnitude of the additional dead space. In both cases, however, tidal volume increased, which may represent a compensatory mechanism for changes in airway resistance [56]. The use of ARDSv devices lengthens the airflow path to the lungs, thereby increasing frictional forces and inspiratory resistance [57]. As a result of six weeks of regular training with increased inspiratory resistance performed for 30 min at an intensity of 70% VO_2_max, an improvement in respiratory muscle strength has been reported [58].

One of the most important consequences of increased CO_2_ partial pressure is its effect on blood pH. Disturbances in pH influence several metabolic reactions in muscle, including glycogenolysis and glycolysis, and affect lactate production, as lactate dissociates into hydrogen and lactate ions. Proper functioning of monocarboxylate transporters (MCT1, MCT4), which mediate lactate transport across the muscle cell membrane, is also determined by systemic acid–base balance [44,59,60,61,62]. A reduction in pH resulting from increased pCO_2_ accumulation is termed respiratory acidosis [44,59,63]. The degree of CO_2_ retention is linearly related to the volume of dead space [52] and indirectly related to the type, intensity, and duration of exercise [44,59], which together determine changes in pH. Respiratory acidosis can inhibit glycolysis and reduce lactate accumulation in the blood. Hypercapnic acidosis, resulting from increased arterial CO_2_ tension (PaCO_2_), may limit the rate of anaerobic glycolysis by reducing the activity of key glycolytic enzymes such as phosphofructokinase, thereby attenuating excessive lactate accumulation during exercise. Elevated CO_2_ and the accompanying decline in pH also alter acid–base balance, modifying both the capacity to buffer hydrogen ions and the recruitment of highly glycolytic muscle fibers. Additional insights into the effects of controlled hypercapnia on metabolic and performance responses have been provided by Ryan et al. [64]. These authors examined how progressively increasing hypercapnia influences exercise parameters in healthy adults. As CO_2_ concentrations rose (breathing either 0%, 2%, or 4% CO2 with 21% oxygen), they observed a systematic decline in blood pH, an increase in minute ventilation, and a clear acceleration in breathing frequency. Despite marked activation of the respiratory center, compensatory mechanisms were insufficient to maintain stable acid–base balance, leading to a reduced time to exhaustion during endurance exercise. At the same time, the authors noted that evolving respiratory acidosis reduced the rate of lactate accumulation, confirming that elevated CO_2_ can modulate anaerobic metabolism by inhibiting intense glycolysis. Kato et al. [44] showed that breathing a gas mixture with 6% CO_2_ during a progressive test, compared with standard breathing conditions, reduced blood pH from 7.18 to 7.12 and lowered lactate concentration from 18 to 12 mmol·L^−1^. However, this modification was not ergogenic, as the participants’ exercise duration decreased by 2 min 20 s. In contrast, Zatoń and Smołka [52] reported that during a 10 min continuous effort at 100 W with 1600 mL of added dead space, blood pH decreased from 7.41 to 7.33 without significant changes in lactate concentration. Similar observations were made by Danek et al. [55] during a single interval session (6 × 10 s sprints with 4 min of active recovery) performed with an ARDSv device of 1200 mL. Moreover, using the same device only during a 10 min warm-up at 60% MAP prior to an identical interval session resulted in reduced lactate concentration (from 4.39 to 3.50 mmol·L^−1^) and a statistically significant decrease in blood pH (from 7.40 to 7.37) [65].

The phenomenon of reduced blood lactate concentration despite a marked decrease in pH may reflect a diminished rate of lactate efflux from muscle to blood [60]. Approximately 80% of lactate transport across the cell membrane depends on the MCT (monocarboxylate transporter) complex and is influenced by hydrogen ion and bicarbonate concentrations on both sides of the membrane, whereas roughly 20% of lactate diffusion occurs along its concentration gradient [66,67,68]. Graham et al. [60] suggested that lower blood lactate concentrations under acidic conditions may be explained by an increased rate of gluconeogenesis. Hollidge-Horvat et al. [66] reported that rising hydrogen ion concentrations slow glycolysis (reducing, among others, pyruvate dehydrogenase activity) and glycogenolysis (lowering glycogen utilization), which may contribute to reduced lactate production. Increased CO_2_ partial pressure (pCO_2_) in the body enhances bicarbonate retention [68,69,70]. This may improve buffering capacity, support pH regulation, and potentially benefit anaerobic metabolic performance, particularly during high-intensity exercise. Accumulation of hydrogen ions due to respiratory or metabolic acidosis impairs normal muscle contractile mechanics [71,72,73]. Factors contributing to this phenomenon include disturbances in strong ion difference (SID) and impaired ATP-dependent Na^+^/K^+^ pump function. High extracellular potassium accumulation reduces muscle fiber excitability and contractility, partly due to the sensitivity of K^+^ channels to changes in H^+^ concentration [74]. Hydrogen ions also compete with calcium ions (Ca^2+^) for binding sites on troponin C, thereby disrupting normal sarcomere function [75,76]. Elevated H^+^ levels can impair Ca^2+^ release and promote faster fatigue development, reducing both the duration and power output of exercise [67,77]. Kato et al. [44] showed that, under hypercapnic conditions during a progressive exercise test, time to exhaustion decreased by 2 min 20 s, from 14:08 to 11:48 (min:s), compared with standard conditions. In contrast, McLellan et al. [63] and Graham et al. [62] reported no differences in test duration when 4% CO_2_ was added to inspired air during a progressive test, compared with normal breathing. Mador et al. [78] demonstrated that 20 min of breathing 8% CO_2_ reduced blood pH to 7.27 and decreased upper-limb muscle contractility without significantly affecting diaphragmatic function. Similarly, Danek et al. [55] found no changes in respiratory muscle strength during interval exercise under conditions of elevated pCO_2_, but observed higher peak power output compared with identical interval exercise without added respiratory load. Thus, exercise under hypercapnic conditions appears to affect respiratory muscle contractility to a lesser extent, which may result from a preferential distribution of blood flow to the diaphragm compared with limb muscles [79].

### 4.1. Respiratory Methods and Devices Provoking Increased CO_2_ Inhalation

Various training and therapeutic strategies that deliberately induce adaptive changes are used to enhance the potential effects of exercise. These strategies include the regulation of inspired gas composition through different forms of breathing manipulation. Woorons et al. [74] employed repeated breath-holding cycles during submaximal exercise to induce hypoxemia, which led to delayed metabolic acidosis and improved exercise performance. Furthermore, physical exercise under hypoxic conditions is thought to cause compensatory vasodilation with a nitric oxide–dependent increase in muscle blood flow [80]. Following this rationale, Lemaître et al. [81] proposed repeated breath-holding as a novel warm-up method that induces tolerable hypoxia. This response occurs in combination with hypercapnia, increased respiratory acidosis, bradycardia, and splenic contraction due to hypoxemia in the initial phase of breath-holding, among other effects [82]. This, in turn, increases the number of circulating erythrocytes, suggesting a potential means of rapidly enhancing the body’s oxygen-transport capacity. Bahenský et al. [83] tested breathing exercises based on the Wim Hof method, which combines deep breathing with breath holds, increases arterial CO_2_ concentrations, and induces hypercapnia.

#### 4.1.1. Training with Voluntary Hypoventilation

The voluntary hypoventilation (VH) method involves intentionally reducing breathing frequency and/or tidal volume during exercise in order to promote CO_2_ accumulation in the blood (hypercapnia) and mild hypoxia. Typically, athletes breathe less frequently and less deeply than normal, often combining this with short breath-hold phases. This technique has been investigated by Woorons and Richalet’s group, pioneers in the study of hypoventilation in sport. Woorons et al. [84] conducted a 4-week training program in which one group of runners performed part of their sessions with voluntary hypoventilation at low lung volumes (after expiration, to accentuate both hypoxia and hypercapnia), while a control group trained identically but breathed normally [74,80]. The outcomes were subtle yet informative: the hypoventilation group did not improve VO_2_max or time to exhaustion compared with controls [81]. However, significant changes in metabolic parameters were observed. After the training period, during submaximal exercise (~90% HR_max), only the athletes training with hypoventilation exhibited less pronounced blood acidosis (higher pH) and higher serum bicarbonate concentrations compared with pre-training values [69,84]. No such changes were noted in the control group. These findings indicate that training with episodes of hypercapnia increased the body’s buffering capacity—muscle and blood became more effective at neutralizing lactic acid produced during intense exercise. The consequence was a smaller decline in blood pH, which may delay the onset of muscle fatigue. Although aerobic capacity itself did not change, the authors suggested that improved buffering could be beneficial for anaerobic performance (e.g., sprinting, interval efforts), where tolerance to acidosis is critical [69,85,86]. This hypothesis has been supported by subsequent studies from the same group, which demonstrated that swimmers and runners training with hypoventilation showed greater improvements in repeated sprint ability or short-distance performance, despite unchanged VO_2_max. In practice, however, voluntary hypoventilation requires caution. It induces not only hypercapnia but also hypoxia (during restricted breathing, arterial oxygen saturation can fall to ~SpO_2_ ≥ 90% or lower) [69,86]. For this reason, it is often compared to altitude or hypoxic training—although, in this case, hypercapnia adds an additional metabolic stimulus. Athletes using this method should be supervised to prevent syncope due to cerebral hypoxia. Nevertheless, a growing body of evidence suggests that well-designed hypoventilation training can improve specific aspects of performance, particularly anaerobic capacity and tolerance to high-intensity exercise. A 2025 meta-analysis [87] pooling results from 10 studies involving trained athletes, examined the effects of repeated-sprint training performed under voluntary hypoventilation over intervention periods ranging from 3 to 6 weeks. Compared with conventional sprint training under normal breathing conditions, hypoventilation-based protocols were associated with a significant improvement in fatigue resistance during repeated sprints, reflected by a smaller decline in sprint speed across successive efforts. In addition, athletes training under hypoventilation consistently exhibited higher post-exercise peak blood lactate concentrations, indicating a greater reliance on anaerobic energy pathways. The direction of these effects suggests that hypoventilation training enhances tolerance to high-intensity metabolic stress rather than improving aerobic capacity [87]. The elevated lactate response was interpreted as evidence of greater activation of anaerobic glycolysis, supporting the notion of adaptations involving enhanced glycogenolytic capacity and glycolytic enzyme activity. Collectively, these findings indicate that exposure to combined hypercapnic–hypoxic stress during sprint training forces skeletal muscle to operate under more demanding metabolic conditions, which over time may increase resistance to sprint-induced fatigue, without translating into improvements in VO_2_max or endurance performance [87].

#### 4.1.2. CO_2_ Inhalation Using Masks That Increase Dead Space

Increasing anatomical dead space has been used to intensify physiological responses and to quantify the magnitude of these changes. To augment additional respiratory dead space volume (ARDSv), tightly fitted respiratory masks covering the nose and mouth have most commonly been employed [52,55,65]. Other devices have also been used, such as “breathing tubes” [88], while in studies involving divers or swimmers, snorkels [89] or custom-designed ARDSv devices enabling mouth-only breathing have been applied [90,91,92]. Breathing through additional dead space volume increases the proportion of CO_2_-enriched air retained within the respiratory tract. Elevated CO_2_ partial pressure arises from mixing inspired air with non-expelled expired air, and the extent of this effect depends on the magnitude of the added dead space [52,65,88]. Increased CO_2_ retention in the respiratory system reduces the concentration gradient between alveolar gas and blood, thereby elevating blood pCO_2_ and inducing a state of hypercapnia [88,89].

An experimental approach involves CO_2_ inhalation through a mask that increases additional respiratory dead space (the so-called ARDS—additional respiratory dead space) during exercise, during recovery intervals between repeated efforts, or during warm-up and post-warm-up phases before physical performance [55,65,90,93]. A mask with a volume of approximately 1000–1200 mL causes a portion of CO_2_-rich expiratory air to be reinhaled, thereby inducing controlled hypercapnia in the athlete. As a result, blood CO_2_ partial pressure rises—an increase in several mmHg above 45 mmHg, consistent with tolerated hypercapnia—leading to mild respiratory acidosis (blood pH < 7.35 with a concomitant increase in bicarbonate concentration) [65]. The body responds by activating the respiratory center: minute ventilation increases through deeper and more frequent breaths [65]. Elevated ventilation, in turn, produces a noticeable rise in oxygen uptake (VO_2_), partly due to the intensified work of the respiratory muscles. Importantly, breathing through an ARDS mask also warms the inspired air and increases body temperature (an increase of ~0.6 °C after warm-up with the mask has been reported) [65]. A slight reduction in arterial oxygen saturation (by approximately 0.9%) has also been observed, which may favor the Bohr effect by facilitating oxygen unloading to the tissues during exercise.

Scientific studies confirm that breathing with added dead space before or during exercise can translate into improved performance parameters. In one of the first experiments [65], healthy men performed a series of interval sprints on a cycle ergometer (Ergomedic Monark 894, Vansbro, Sweden), breathing through a 1200 mL ARDS mask in one session and breathing normally in another. Total work performed during sprints with the ARDS mask was ~4.4% higher than without the mask; mean power output was also significantly higher (772 ± 148 W vs. 750 ± 139 W), and peak power increased by approximately 8% compared with conventional warm-up (conference data). The more intensive breathing response resulted in a markedly higher minute ventilation (+13% compared with control conditions) and about 31% greater oxygen uptake during interval exercise with the mask [65]. Despite this heightened physiological response, no increase in perceived exertion (RPE) or respiratory muscle fatigue was reported—subjective exercise intensity was similar to that without the mask. Furthermore, under hypercapnic conditions, greater internal acidosis was observed (mean post-exercise blood pH with ARDS decreased to 7.26 vs. 7.29 without the mask), alongside higher blood HCO_3_^−^ concentrations (+7.6% vs. control). This response suggests enhanced buffering and metabolic processes that may favor phosphocreatine resynthesis and delay lactate accumulation in muscle. The authors concluded that breathing CO_2_-enriched air elicits a range of physiological reactions that are advantageous for high-intensity exercise, without increasing subjective discomfort.

Using an ARDS mask during warm-up prior to the main exercise bout also yields measurable benefits. Danek et al. [65] conducted a study in which a standard warm-up preceding interval sprints was extended by a 10 min period of breathing through a 1200 mL mask. Athletes prepared in this way performed more total work (+2.9%) and achieved higher mean power during the subsequent exercise compared with a control trial without the mask. They also exhibited elevated end-tidal pCO_2_ (~45 vs. 42 mmHg without the mask) and higher body temperature after warm-up, indicating sustained muscle vasodilation and improved perfusion of working muscles. Hebisz et al. [93] showed that breathing through a 1000 mL mask during the interval between warm-up and a cycling time trial helped maintain the “warm-up effect.” After an 8 min passive break, cyclists using ARDS displayed significantly higher VO_2_ in the subsequent effort (VO_2_peak ~4.22 L·min^−1^ with the mask vs. 3.98 L·min^−1^ without) and reported a lower respiratory exchange ratio (RER) and reduced perceived exertion (RPE 18.0 vs. 18.9) compared with normal breathing. These findings suggest that ARDS-induced hypercapnia prevents the decay of warm-up–induced adaptations—likely by maintaining capillary vasodilation and muscle oxygenation—thereby enabling more intense exercise at a lower subjective cost.

Similar results have been obtained in swimming. Danek et al. [90] observed that sprint swimmers achieved faster 50 m times (~1.2% improvement) when they performed an additional land-based warm-up with ARDS breathing between their in-water warm-up and the race start. In these swimmers, post–hypercapnic warm-up blood pCO_2_ reached ~49.7 mmHg (vs. ~40 mmHg in standard conditions), confirming the induction of tolerated hypercapnia. The authors emphasized that a 20 min post-warm-up phase with the mask maintained elevated respiratory and metabolic parameters up to the start, which translated into improved performance.

In summary, CO_2_ inhalation using a mask that increases dead space represents an innovative warm-up support technique. Studies consistently indicate its ergogenic effects: increased oxygen uptake and ventilation, maintained high muscle perfusion, and a slight reduction in blood pH with enhanced buffering capacity all contribute to improved anaerobic performance. Athletes using such hypercapnic warm-up protocols achieve higher peak and mean power outputs, perform more work during sprints, and show improved time-trial performance in speed-oriented events. Importantly, these benefits are achieved without a rise in perceived exertion—breathing through additional dead space is perceived similarly to normal breathing, despite clearly intensified physiological responses. This technique therefore extends the duration of the warm-up effect and allows athletes to fully exploit it at the start of competition by preserving favorable adaptations that would otherwise dissipate during the pre-competition interval. Current findings suggest substantial potential for ARDS mask use in speed–endurance sports; however, further studies are necessary to fully elucidate the underlying mechanisms, optimize protocols, and evaluate long-term training effects.

**Table 1:** Studies included in review.

Key Findings/Relevance	Intervention (vs. Control)	Sport/Population	Study
↑ cortisol (~35–40%) and autonomic arousal; strong HPA-axis activation, no performance relevance	7.5% CO_2_ inhalation (vs. air)	Healthy adults	Kaye et al., 2004 [11]
Reliable induction of panic-like responses; laboratory panic model	35% CO_2_ inhalation (vs. air)	Adults (clinical + controls)	Coryell & Arndt, 1999 [22]
Dose-dependent ↑ anxiety and negative affect; higher reactivity in older subjects	7.5% CO_2_ inhalation (vs. air)	Healthy volunteers	Griez et al., 2007 [21]
Impaired cognitive performance; ↑ perceived mental workload	7.5% CO_2_ during cognitive tasks (vs. air)	Healthy adults	Diaper et al., 2012 [23]
Possible impairment of cerebrovascular regulation and cognition (review evidence)	Chronic mild hypercapnia (vs. normocapnia)	Confined-environment personnel	Carr et al., 2025 [14]
↑ sleepiness and ↓ cognitive performance at work	Indoor CO_2_ exposure (vs. lower CO_2_)	Office workers	Vehviläinen et al., 2016 [34]
Metabolic stress and ↓ task performance at 1000–3000 ppm CO_2_	Indoor CO_2_ exposure (vs. ~400 ppm)	Indoor populations	Azuma et al., 2018 [10]
↑ respiratory acidosis, ↓ time to exhaustion despite ↓ lactate	Exercise with 6% CO_2_ (vs. normocapnia)	Healthy adults	Kato et al., 2005 [44]
Altered glycolytic flux; ↓ lactate via acidosis-mediated inhibition	Hypercapnic exercise (vs. normocapnia)	Healthy adults	Ehrsam et al., 1982 [45]
↑ muscle lactate release and glycolytic intermediates	CO_2_ + exercise (vs. normocapnia)	Healthy adults	Graham et al., 1986 [60]
Modified lactate response; no endurance benefit	Hypercapnic incremental exercise (vs. normocapnia)	Healthy adults	McLellan, 1991 [63]
↑ HR, BP and cerebral perfusion during orthostatic stress	5% CO_2_ breathing (vs. air)	Healthy adults	Howden et al., 2004 [28]
↑ ventilatory demand; ↓ respiratory performance	Moderate CO_2_ during exercise (vs. lower CO_2_)	Healthy adults	Mishra et al., 2021 [29]
↑ cerebral blood flow at rest and exercise onset; no sustained performance gain	Hypercapnia (vs. normocapnia)	Healthy adults	Ogoh et al., 2009 [48]
↑ VE and altered breathing pattern at maximal exercise	Added dead space (vs. none)	Healthy adults	McParland et al., 1991 [54]
Altered ventilatory and circulatory responses; no endurance improvement	Dead space during exercise (vs. none)	Healthy adults	Zatoń & Smołka, 2011 [52]
Potentiated ventilatory response to exercise	CO_2_/dead-space loading (vs. normal breathing)	Healthy adults	Poon, 1992 [56]
↑ buffering capacity; no change in VO_2_max	Voluntary hypoventilation training (vs. normal breathing)	Trained runners/swimmers	Woorons et al., 2008 [84]
↑ repeated-sprint fatigue resistance; no aerobic adaptations	Repeated sprints with VH (vs. normal breathing)	Trained athletes	Woorons et al., 2010 [69]
Improved short-distance performance only	VH during running (vs. conventional training)	Runners	Prieur et al., 2006 [86]
↑ total sprint work (~+4.4%) and mean power; RPE unchanged	Sprint intervals + ARDS (vs. no ARDS)	Healthy active men	Danek et al., 2020 [55]
Improved sprint-interval performance and buffering	Warm-up + ARDS (vs. standard warm-up)	Trained cyclists	Danek & Zatoń, 2022 [65]
Preservation of VO_2_ and reduced fatigue post warm-up	ARDS during break (vs. passive rest)	Competitive cyclists	Hebisz et al., 2025 [93]
↑ 50 m sprint performance (~1–2%)	Re-warm-up + ARDS (vs. standard)	Sprint swimmers	Danek et al., 2025 [90]
Feasible respiratory muscle loading; no major adverse effects	Tube breathing (vs. normal breathing)	Healthy volunteers	Koppers et al., 2006 [88]
↑ CO_2_ rebreathing; altered ventilatory and metabolic responses	Snorkel rebreathing (vs. normal breathing)	Divers/swimmers	Toklu et al., 2003 [89]
↑ CO_2_ tolerance; performance effects uncertain	Breath-hold breathing (vs. conventional training)	Adolescent/endurance athletes	Bahenský et al., 2020 [83]

Note: ↑—increase; ↓—decrease ARDS—additional respiratory dead space; BP—blood pressure; CO_2_—carbon dioxide; HPA axis—hypothalamic–pituitary–adrenal axis; HR—heart rate; RPE—rating of perceived exertion; VE—minute ventilation; VO_2_—oxygen uptake; VH—voluntary hypoventilation.

## 5. Safety and Contraindications

### 5.1. Populations in Whom Hypercapnia-Inducing Methods Should Be Avoided

Hypercapnia-inducing methods (including CO_2_ inhalation, CO_2_ rebreathing via increased respiratory dead space, and voluntary hypoventilation) should be avoided in individuals with cardiovascular disease (e.g., ischemic heart disease, heart failure, or known arrhythmias), respiratory disorders (e.g., asthma or chronic obstructive pulmonary disease), uncontrolled hypertension, impaired cerebrovascular regulation, or a history of syncope or autonomic instability. Caution is also warranted in individuals with panic disorder, severe anxiety, or heightened sensitivity to dyspnea, as CO_2_ exposure is known to provoke panic-like responses.

### 5.2. Warning Symptoms Requiring Immediate Cessation

Any application of hypercapnia-inducing methods should be immediately discontinued if syncope or presyncope (e.g., dizziness, visual disturbances), chest pain, palpitations, severe dyspnea, panic symptoms, marked confusion, cognitive impairment, or loss of motor control occur.

### 5.3. General Monitoring and Safety Principles

Hypercapnia-inducing methods should not be performed alone and require appropriate supervision. Participants should be continuously observed for signs of physiological or psychological distress. Any applied use should prioritize conservative exposure, immediate termination upon symptom onset, and avoidance of competitive or peer-pressure environments. These methods should be regarded as experimental and are not substitutes for established training strategies.

### 5.4. Clinical Versus Non-Clinical Exposure

Exposure to CO_2_-enriched gas mixtures (e.g., carbogen) in medical or research settings occurs exclusively under controlled clinical conditions with predefined safety protocols and continuous monitoring. Such exposure should not be extrapolated to unsupervised training, commercial devices, or non-medical performance contexts.

## 6. Knowledge Gaps and Directions for New Research

Despite promising evidence that controlled, short-term hypercapnia can elicit distinct physiological responses with potential ergogenic effects—such as enhanced buffering capacity, altered lactate kinetics, increased ventilation, and elevated oxygen uptake—the current body of research is fragmented and insufficient to establish clear long-term safety or performance recommendations. Most available studies assess only acute responses, often involving small samples, short protocols, and limited physiological endpoints. As a result, several critical gaps in knowledge remain. First, long-term adaptations to repeated hypercapnic exposure are poorly understood. Existing studies focus primarily on single sessions or short training blocks, leaving unanswered questions about the durability of metabolic, respiratory, or neuromuscular adaptations, as well as potential changes in chemosensitivity or respiratory muscle load. Second, the multisystem effects of hypercapnia require comprehensive investigation. While evidence suggests that CO_2_ influences not only ventilation and acid–base balance but also cognitive function, stress responses, immune signaling, and vascular regulation, no studies have simultaneously evaluated these systems during hypercapnic training. Understanding whole-body consequences is essential for determining the true safety profile of such interventions. Third, substantial interindividual variability in CO_2_ sensitivity—including differences in dyspnea tolerance, anxiety reactivity, and chemoreflex responsiveness—may significantly affect both safety and performance outcomes. Identifying predictors of tolerance and adverse responses is crucial before hypercapnic methods can be widely applied. Safety concerns in applied sport settings remain unresolved. Although controlled mild hypercapnia appears safe under laboratory supervision, risks such as syncope during breath-hold practices, arrhythmias at higher CO_2_ loads, impaired cognitive performance, or compromised immune function with repeated exposure have not been adequately examined in longer-term or real-world contexts. Protocols used to induce hypercapnia lack standardization. Current approaches vary widely in CO_2_ intensity, duration, device type, timing relative to exercise (warm-up, intervals, recovery), and training frequency. Without consistent methodology, determining dose–response relationships and optimal training strategies remains impossible. Finally, mechanistic understanding of the ergogenic potential of hypercapnia is incomplete. While some studies demonstrate improved tolerance to high-intensity exercise or altered lactate dynamics, the underlying mechanisms—such as changes in enzyme activity, ion transport, muscle oxygenation, or fiber recruitment—require further targeted research.

In summary, future research should integrate physiological, immunological, neurocognitive, and clinical approaches to define both the safety boundaries and the potential performance benefits of hypercapnia-based training. Only comprehensive, long-term studies can determine whether controlled CO_2_ inhalation represents a viable training method or whether its use should remain limited to experimental and supervised conditions.

## 7. Conclusions

It is increasingly evident that carbon dioxide (CO_2_) is more than a mere by-product of cellular metabolism. Rather, it should be regarded as a potent biological effector with both protective and potentially harmful actions. Importantly, it remains unclear to what extent hypercapnia should be tolerated as an adaptive phenomenon, and when it should be prevented or treated using invasive extracorporeal CO_2_ removal techniques. In healthy individuals, CO_2_ inhalation carries a substantial risk of immediate adverse effects, including intense dyspnea, panic-like anxiety, cognitive impairment, and, at higher concentrations, loss of consciousness. Even relatively low concentrations (approximately 0.5–3% CO_2_) can negatively affect comfort and exercise capacity, leading to earlier onset of fatigue and reduced performance [10]. Although repeated exposure to moderate hypercapnia—such as during voluntary hypoventilation or added dead-space breathing—may enhance buffering capacity and tolerance to high-intensity exercise, these effects are context-specific and do not translate into improvements in aerobic capacity or endurance performance. Importantly, the use of CO_2_-based devices or breathing strategies outside controlled and supervised settings cannot be recommended. Commercial or unsupervised application of training masks or deliberate CO_2_ rebreathing poses potential health risks and lacks sufficient scientific justification. At present, the risks associated with CO_2_ inhalation outweigh its potential benefits for performance enhancement in healthy populations. Hypercapnia-based methods should therefore remain confined to experimental and carefully monitored conditions until robust evidence on long-term safety and efficacy becomes available. Hypercapnia can have serious health consequences if it is too severe or prolonged. Ongoing medical research continues to explore both the detrimental effects of elevated CO_2_ (e.g., in urban indoor environments with inadequate ventilation) and its potential therapeutic actions (such as anti-inflammatory or anticonvulsant effects of 5% CO_2_). However, in healthy individuals, any such interventions must be approached with great caution.

In summary, based on current knowledge, the risks associated with CO_2_ inhalation in healthy people outweigh the potential benefits for performance enhancement. This does not mean that the concept of “hypercapnic training” is entirely without merit—some findings are indeed promising. Nonetheless, further research is required to define optimal protocols, safe ranges of hypercapnia, and the long-term consequences of such interventions. Until then, CO_2_ should be regarded primarily as a risk factor—a gas whose excess we seek to avoid rather than deliberately inhale when the goal is health and physical performance.

## Data Availability

No new data were created or analyzed in this study.

## References

[1] Patel N., Sharma S. (2023). Respiratory Acidosis. StatPearls.

[2] Spector S., McKhann C.F. (1948). Respiratory acidosis and alkalosis in children. J. Pediatr..

[3] Ainslie P.N., Duffin J. (2009). Integration of cerebrovascular CO_2_ reactivity and chemoreflex control of breathing: Mechanisms of regulation, measurement, and interpretation. Am. J. Physiol. Regul. Integr. Comp. Physiol..

[4] Teboul J.L., Scheeren T. (2017). Understanding the Haldane effect. Intensive Care Med..

[5] Cummins E.P., Selfridge A.C., Sporn P.H., Sznajder J.I., Taylor C.T. (2014). Carbon dioxide-sensing in organisms and its implications for human disease. Cell. Mol. Life Sci..

[6] Osorio-Rodríguez E., Correa-Guerrero J., Rodelo-Barrios D., Bonilla-Llanos M., Rebolledo-Maldonado C., Patiño-Patiño J., Viera-Torres J., Arias-Gómez M., Gracia-Ordoñez M., González-Betancur D. (2025). Hypercapnia as a double-edged modulator of innate immunity and alveolar epithelial repair: A PRISMA-ScR scoping review. Int. J. Mol. Sci..

[7] Nattie E. (1999). CO_2_, brainstem chemoreceptors and breathing. Prog. Neurobiol..

[8] Guyenet P.G. (2014). Regulation of breathing and autonomic outflows by chemoreceptors. Compr. Physiol..

[9] Liu J.J.W., Ein N., Gervasio J., Vickers K. (2019). Subjective and physiological responses to the 35% carbon dioxide challenge in healthy and non-clinical control populations: A meta-analysis and systematic review. Anxiety Stress Coping.

[10] Azuma K., Kagi N., Yanagi U., Osawa H. (2018). Effects of low-level inhalation exposure to carbon dioxide in indoor environments: A short review on human health and psychomotor performance. Environ. Int..

[11] Kaye J., Buchanan F., Kendrick A., Johnson P., Lowry C., Bailey J., Nutt D., Lightman S. (2004). Acute carbon dioxide exposure in healthy adults: Evaluation of a novel means of investigating the stress response. J. Neuroendocrinol..

[12] van Duinen M.A., Schruers K.R., Maes M., Griez E.J. (2007). CO_2_ challenge induced HPA axis activation in panic. Int. J. Neuropsychopharmacol..

[13] Jacobson T.A., Kler J.S., Hernke M.T., Braun R.K., Meyer K.C., Funk W.E. (2019). Direct human health risks of increased atmospheric carbon dioxide. Nat. Sustain..

[14] Carr J.M.J.R., Ainslie P.N., Day T. (2025). Confined spaces in space: Cerebral implications of chronic elevations of inspired carbon dioxide and implications for long-duration space travel. Exp. Physiol..

[15] Hirotsu C., Tufik S., Andersen M.L. (2015). Interactions between sleep, stress, and metabolism. Sleep Sci..

[16] Salcedo-Betancourt J.D., Moe O.W. (2024). The effects of acid on calcium and phosphate metabolism. metabolism. Int. J. Mol. Sci..

[17] Van Duinen M.A., Schruers K.R., Maes M., Griez E.J. (2005). CO_2_ challenge results in hypothalamic-pituitary-adrenal activation in healthy volunteers. J. Psychopharmacol..

[18] Xu F., Uh J., Brier M.R., Hart J., Lu H. (2011). The influence of carbon dioxide on brain activity and metabolism in conscious humans. J. Cereb. Blood Flow Metab..

[19] Zhang Y., Liu Z., Chen W., Liang J., Wang J. (2011). Magnetic resonance imaging of vascular oxygenation with carbogen (95% O_2_ 5% CO_2_). Investig. Ophthalmol. Vis. Sci..

[20] Perna G., Battaglia M., Garberi A., Arancio C., Bertani A., Bellodi L. (1994). Carbon dioxide/oxygen challenge test in panic disorder. Psychiatry Res..

[21] Griez E.J., Colasanti A., van Diest R., Salamon E., Schruers K. (2007). Carbon dioxide inhalation induces dose-dependent and age-related negative affectivity. PLoS ONE.

[22] Coryell W., Arndt S. (1999). The 35% CO_2_ inhalation procedure: Test-retest reliability. Biol. Psychiatry.

[23] Diaper A., Nutt D.J., Munafò M.R., White J.L., Farmer E.W., Bailey J.E. (2012). The effects of 7.5% carbon dioxide inhalation on task performance in healthy volunteers. J. Psychopharmacol..

[24] Schneberger D., Cloonan D., DeVasure J.M., Bailey K.L., Romberger D.J., Wyatt T.A. (2015). Effect of elevated carbon dioxide on bronchial epithelial innate immune receptor response to organic dust from swine confinement barns. Int. Immunopharmacol..

[25] Casalino-Matsuda S.M., Wang N., Ruhoff P.T., Matsuda H., Nlend M.C., Nair A., Szleifer I., Beitel G.J., Sznajder J.I. (2018). Hypercapnia alters expression of immune response genes in airway epithelial cells. Sci. Rep..

[26] Billert H., Bednarek E., Kusza K., Ponichter M., Kurpisz M. (2021). Effect of acute isooxic hypercapnia on oxidative activity of systemic neutrophils in endotoxemic rabbits. Cent. Eur. J. Immunol..

[27] Gałgańska H., Jarmuszkiewicz W., Gałgański Ł. (2023). Carbon dioxide and MAPK signalling: Towards therapy for inflammation and related processes. Cell. Mol. Biol. Lett..

[28] Howden R., Roddie I.C., Wallace A.M. (2004). The effects of breathing 5% carbon dioxide on human cardiovascular responses and tolerance to orthostatic stress. Exp. Physiol..

[29] Mishra A.K., Schiavon S., Wargocki P., Tham K.W. (2021). Respiratory performance of humans exposed to moderate levels of carbon dioxide. Indoor Air.

[30] Garner M., Attwood A., Baldwin D.S., James A., Munafò M.R. (2011). Inhalation of 7.5% carbon dioxide increases threat processing in humans. Neuropsychopharmacology.

[31] Bailey J.E., Argyropoulos S.V., Kendrick A.H., Nutt D.J. (2005). Behavioral and cardiovascular effects of 7.5% CO_2_ in human volunteers. Depress. Anxiety.

[32] Almanza-Hurtado A., Polanco Guerra C., Martínez-Ávila M.C., Borré-Naranjo D., Rodríguez-Yanez T., Dueñas-Castell C. (2022). Hypercapnia from physiology to practice. Int. J. Clin. Pract..

[33] Satish U., Mendell M.J., Shekhar K., Hotchi T., Sullivan D., Streufert S., Fisk W.J. (2012). Is CO_2_ an indoor pollutant? Direct effects of low-to-moderate carbon dioxide concentrations on human decision-making performance. Environ. Health Perspect..

[34] Vehviläinen T., Lindholm H., Rintamäki H., Pääkkönen R., Hirvonen A., Niemi O., Vinha J. (2016). High indoor CO_2_ concentrations in an office environment increases the transcutaneous CO_2_ level and sleepiness during cognitive work. J. Occup. Environ. Hyg..

[35] Battisti-Charbonney A., Fisher J., Duffin J. (2011). The cerebrovascular response to carbon dioxide in humans. J. Physiol..

[36] Morales-Quinteros L., Camprubí-Rimblas M., Bringué J., Bos L.D., Schultz M.J., Artigas A. (2019). The role of hypercapnia in acute respiratory failure. Intensive Care Med. Exp..

[37] Bruce R.M., White M.J. (2015). The ventilatory response to muscle afferent activation during concurrent hypercapnia in humans: Central and peripheral mechanisms. Exp. Physiol..

[38] Nattie E., Li A. (2012). Central chemoreceptors: Locations and functions. Compr. Physiol..

[39] Greiner J.G., Clegg M.E., Walsh M.L., White M.D. (2010). No effect of skin temperature on human ventilation response to hypercapnia during light exercise with a normothermic core temperature. Eur. J. Appl. Physiol..

[40] McGurk S.P., Blanksby B.A., Anderson M.J. (1995). The relationship between carbon dioxide sensitivity and sprint or endurance performance in young swimmers. Br. J. Sports Med..

[41] Shigemura M., Welch L.C., Sznajder J.I. (2020). Hypercapnia regulates gene expression and tissue function. Front. Physiol..

[42] Ohkuwa T., Fujitsuka N., Utsuno T., Miyamura M. (1980). Ventilatory response to hypercapnia in sprint and long-distance swimmers. Eur. J. Appl. Physiol. Occup. Physiol..

[43] Miyamoto T., Inagaki M., Takaki H., Kawada T., Shishido T., Kamiya A., Sugimachi M. (2012). Adaptation of the respiratory controller contributes to the attenuation of exercise hyperpnea in endurance-trained athletes. Eur. J. Appl. Physiol..

[44] Kato T., Tsukanaka A., Harada T., Kosaka M., Matsui N. (2005). Effect of hypercapnia on changes in blood pH, plasma lactate and ammonia due to exercise. Eur. J. Appl. Physiol..

[45] Ehrsam R.E., Heigenhauser G.J., Jones N.L. (1982). Effect of respiratory acidosis on metabolism in exercise. J. Appl. Physiol. Respir. Environ. Exerc. Physiol..

[46] Jones N.L., Sutton J.R., Taylor R., Toews C.J. (1977). Effect of pH on cardiorespiratory and metabolic responses to exercise. J. Appl. Physiol. Respir. Environ. Exerc. Physiol..

[47] Frydrychowski A.F., Wszedybyl-Winklewska M., Guminski W., Lass P., Bandurski T., Winklewski P.J. (2012). Effects of acute hypercapnia on the amplitude of cerebrovascular pulsation in humans registered with a non-invasive method. Microvasc. Res..

[48] Ogoh S., Ainslie P.N., Miyamoto T. (2009). Onset responses of ventilation and cerebral blood flow to hypercapnia in humans: Rest and exercise. J. Appl. Physiol..

[49] Wood H.E., Mitchell G.S., Babb T.G. (2008). Short-term modulation of the exercise ventilatory response in young men. J. Appl. Physiol..

[50] Kumar P., Prabhakar N.R. (2012). Peripheral chemoreceptors: Function and plasticity of the carotid body. Compr. Physiol..

[51] Kumar P., Bin-Jaliah I. (2007). Adequate stimuli of the carotid body: More than an oxygen sensor?. Respir. Physiol. Neurobiol..

[52] Zatoń M., Smołka Ł. (2011). Circulatory and respiratory response to exercise with added respiratory dead space. Hum. Mov..

[53] Wood H.E., Mitchell G.S., Babb T.G. (2009). Breathing mechanics during exercise with added dead space reflect mechanisms of ventilatory control. Respir. Physiol. Neurobiol..

[54] McParland C., Mink J., Gallagher C.G. (1991). Respiratory adaptations to dead space loading during maximal incremental exercise. J. Appl. Physiol..

[55] Danek N., Michalik K., Smolarek M., Zatoń M. (2020). Acute effects of using added respiratory dead space volume in a cycling sprint interval exercise protocol: A cross-over study. Int. J. Environ. Res. Public Health.

[56] Poon C.S. (1992). Potentiation of exercise ventilatory response by airway CO_2_ and dead space loading. J. Appl. Physiol..

[57] Goodarzi-Ardakani V., Taeibi-Rahni M., Salimi M.R., Ahmadi G. (2016). Computational simulation of temperature and velocity distribution in human upper respiratory airway during inhalation of hot air. Respir. Physiol. Neurobiol..

[58] McEntire S.J., Smith J.R., Ferguson C.S., Brown K.R., Kurti S.P., Harms C.A. (2016). The effect of exercise training with an additional inspiratory load on inspiratory muscle fatigue and time-trial performance. Respir. Physiol. Neurobiol..

[59] Ostergaard L., Kjaer K., Jensen K., Gladden L.B., Martinussen T., Pedersen P.K. (2012). Increased steady-state VO_2_ and larger O_2_ deficit with CO_2_ inhalation during exercise. Acta Physiol..

[60] Graham T.E., Barclay J.K., Wilson B.A. (1986). Skeletal muscle lactate release and glycolytic intermediates during hypercapnia. J. Appl. Physiol..

[61] Cabrera M.E., Saidel G.M., Kalhan S.C. (1999). Lactate metabolism during exercise: Analysis by an integrative systems model. Am. J. Physiol..

[62] Graham T.E., Wilson B.A., Sample M., Van Dijk J., Goslin B. (1982). The effects of hypercapnia on the metabolic response to steady-state exercise. Med. Sci. Sports Exerc..

[63] McLellan T.M. (1991). The influence of a respiratory acidosis on the exercise blood lactate response. Eur. J. Appl. Physiol. Occup. Physiol..

[64] Ryan B.J., Seeley A.D., Pitsas D.M., Mayer T.A., Caldwell A.R., Ceaser T.G., Luippold A.J., Charkoudian N., Salgado R.M. (2022). Influence of graded hypercapnia on endurance exercise performance in healthy humans. Am. J. Physiol. Regul. Integr. Comp. Physiol..

[65] Danek N., Michalik K., Zatoń M. (2022). Warm-up with added respiratory dead space volume mask improves the performance of the cycling sprint interval exercise: Cross-over study. Front. Physiol..

[66] Hollidge-Horvat M.G., Parolin M.L., Wong D., Jones N.L., Heigenhauser G.J.F. (2000). Effect of induced metabolic alkalosis on human skeletal muscle metabolism during exercise. Am. J. Physiol. Endocrinol. Metab..

[67] Hirche H.J., Hombach V., Langohr H.D., Wacker U., Busse J. (1975). Lactic acid permeation rate in working gastrocnemii of dogs during metabolic alkalosis and acidosis. Pflugers Arch..

[68] Oppersma E., Doorduin J., van der Hoeven J.G., Veltink P.H., van Hees H.W.H., Heunks L.M.A. (2018). The effect of metabolic alkalosis on the ventilatory response in healthy subjects. Respir. Physiol. Neurobiol..

[69] Woorons X., Gamelin F.X., Lamberto C., Pichon A., Richalet J.P. (2010). Repeated sprint ability with voluntary hypoventilation. Int. J. Sports Physiol. Perform..

[70] Woorons X., Mollard P., Pichon A., Duvallet A., Richalet J.P., Lamberto C. (2007). Prolonged expiration down to residual volume leads to severe arterial hypoxemia in athletes during submaximal exercise. Respir. Physiol. Neurobiol..

[71] Woodward M., Debold E.P. (2018). Acidosis and phosphate directly reduce myosin’s force-generating capacity through distinct molecular mechanisms. Front. Physiol..

[72] Debold E.P., Beck S.E., Warshaw D.M. (2008). Effect of low pH on single skeletal muscle myosin mechanics and kinetics. Am. J. Physiol. Cell Physiol..

[73] Westerblad H., Bruton J.D., Lännergren J. (1997). The effect of intracellular pH on contractile function of intact, single fibres of mouse muscle declines with increasing temperature. J. Physiol..

[74] Street D., Nielsen J.J., Bangsbo J., Juel C. (2005). Metabolic alkalosis reduces exercise-induced acidosis and potassium accumulation in human skeletal muscle interstitium. J. Physiol..

[75] Allen D.G. (2009). Fatigue in working muscles. J. Appl. Physiol..

[76] Ortenblad N., Lunde P.K., Levin K., Andersen J.L., Pedersen P.K. (2000). Enhanced sarcoplasmic reticulum Ca^2+^ release following intermittent sprint training. Am. J. Physiol. Regul. Integr. Comp. Physiol..

[77] Ueno S., Yokoyama K., Nakagawa M., Araki S. (2002). Effects of pH and temperature on force and stiffness of skeletal muscle fibers during contraction and relaxation in relation to musculoskeletal disorders. Ind. Health.

[78] Mador M.J., Wendel T., Kufel T.J. (1997). Effect of acute hypercapnia on diaphragmatic and limb muscle contractility. Am. J. Respir. Crit. Care Med..

[79] Harms C.A., Babcock M.A., McClaran S.R., Pegelow D.F., Nickele G.A., Nelson W.B., Dempsey J.A. (1997). Respiratory muscle work compromises leg blood flow during maximal exercise. J. Appl. Physiol..

[80] Casey D.P., Joyner M.J. (2012). Compensatory vasodilatation during hypoxic exercise: Mechanisms responsible for matching oxygen supply to demand. J. Physiol..

[81] Lemaître F., Joulia F., Chollet D. (2010). Apnea: A new training method in sport?. Med. Hypotheses.

[82] Baković D., Valic Z., Eterović D., Vukovic I., Obad A., Marinović-Terzić I., Dujić Z. (2003). Spleen volume and blood flow response to repeated breath-hold apneas. J. Appl. Physiol..

[83] Bahenský P., Bunc V., Tlustý P., Grosicki G.J. (2020). Effect of an eleven-day altitude training program on aerobic and anaerobic performance in adolescent runners. Medicina.

[84] Woorons X., Mollard P., Pichon A., Duvallet A., Richalet J.P. (2008). Effects of voluntary hypoventilation on blood gases and acid–base balance during exercise. Eur. J. Appl. Physiol..

[85] Woorons X., Pichon A., Lamberto C., Duvallet A., Richalet J.P. (2008). Influence of different voluntary hypoventilation techniques on tolerance to high-intensity exercise. Int. J. Sports Med..

[86] Prieur F., Busso T., Castells J., Bonnefoy R. (2006). Running performance and respiratory variables in hypoventilation training. Eur. J. Appl. Physiol..

[87] Précart C., Bouten J., Woorons X., Fornasier-Santos C., Millet G.P., Brocherie F. (2025). Repeated-sprint training in hypoxia induced by voluntary hypoventilation at low lung volume: A meta-analysis. Sports Med. Open.

[88] Koppers R.J., Vos P.J., Folgering H.T. (2006). Tube breathing as a new potential method to perform respiratory muscle training: Safety in healthy volunteers. Respir. Med..

[89] Toklu A.S., Kayserilioğlu A., Unal M., Ozer S., Aktaş S. (2003). Ventilatory and metabolic response to rebreathing the expired air in the snorkel. Int. J. Sports Med..

[90] Danek N., Szczepan S., Wróblewska Z., Michalik K., Zatoń M. (2025). Hypercapnic warm-up and re-warm-up—A novel experimental approach in swimming sprint. PLoS ONE.

[91] Szczepan S., Pożarowszczyk-Kuczko B., Michalik K. (2025). Validity of 2-point method for load-velocity profiling in free swimming, with snorkel, and with added respiratory dead space mask. Sci. Rep..

[92] Szczepan S., Danek N., Michalik K., Wróblewska Z., Zatoń M. (2020). Influence of a six-week swimming training with added respiratory dead space on respiratory muscle strength and pulmonary function in recreational swimmers. Int. J. Environ. Res. Public Health.

[93] Hebisz P., Hebisz R., Danek N. (2025). Prolonging the warm-up effect by using additional respiratory dead space volume after the cessation of warm-up exercise. J. Clin. Med..

